# Characterization of familial hypercholesterolemia in Taiwanese ischemic stroke patients

**DOI:** 10.18632/aging.203320

**Published:** 2021-07-27

**Authors:** Hsin Tung, Hsueh-Ju Lin, Po-Lin Chen, Tsai-Jung Lu, Pei-Pei Jhan, Jun-Peng Chen, Yi-Ming Chen, Chen-Chin Wu, Yung-Yang Lin, Tzu-Hung Hsiao

**Affiliations:** 1 Institute of Clinical Medicine, National Yang Ming Chiao Tung University, Taipei, Taiwan; 2 Center of Faculty Development, Taichung Veterans General Hospital, Taichung, Taiwan; 3 Division of Epilepsy, Neurological Institute, Taichung Veterans General Hospital, Taichung, Taiwan; 4 Department of Medical Research, Taichung Veterans General Hospital, Taichung, Taiwan; 5 Division of General Neurology, Neurological Institute, Taichung Veterans General Hospital, Taichung, Taiwan; 6 School of Medicine, National Yang-Ming Chiao Tung University, Taipei, Taiwan; 7 Biostatistics Task Force of Taichung Veterans General Hospital, Taichung, Taiwan; 8 Division of Allergy, Immunology and Rheumatology, Taichung Veterans General Hospital, Taichung, Taiwan; 9 Department of Critical Care Medicine, Taipei Veterans General Hospital, Taipei, Taiwan; 10 Institute of Brain Science, National Yang Ming Chiao Tung University, Taipei, Taiwan; 11 Department of Public Health, Fu Jen Catholic University, New Taipei City, Taiwan; 12 Institute of Genomics and Bioinformatics, National Chung Hsing University, Taichung, Taiwan

**Keywords:** familial hypercholesterolemia, ischemic stroke, carotid intima-media thickness, atherosclerosis, low-density lipoprotein cholesterol

## Abstract

Familial hypercholesterolemia (FH) is a common genetic disorder characterized by a lifelong elevated low-density lipoprotein cholesterol (LDL-C) level. The relationship between FH and ischemic stroke is still controversial. We enrolled ischemic stroke patients prospectively in our neurological ward, and divided them into two groups according to LDL-C levels with a threshold of 130 mg/dl. Targeted sequencing was performed in all stroke patients for *LDLR, APOB*, and *PCSK9* genes. The fifty-eight high-LDL subjects were older, prevalence of previous myocardial infarction/stroke history was lower, and the first stroke age was older compared with values in the sixty-three low-LDL cases. The prevalence of FH in Han-Chinese stroke patients was 5.0%, and was 10.3% in those with a higher LDL-C level. We identified six carriers, who had higher percentages of large vessel stroke subtype (66.7% vs. 15.4%) and transient ischemic attack (33.3% vs. 3.8%), previous myocardial infarction/stroke history (50.0% vs. 11.5%), statin use (50.0% vs. 11.5%), and increased carotid intima-media thickness (IMT) (0.9-1.2mm vs.0.7-9.0mm) compared with the other hypercholesterolemic patients without pathogenic variants. Ischemic stroke patients carrying FH pathogenic variants seemed to have a higher risk for large artery stroke and transient ischemic attack. The IMT exam could be useful to screen for FH in hypercholesterolemic stroke patients.

## INTRODUCTION

Familial hypercholesterolemia (FH) is the most common monogenic disorder with an autosomal dominant hereditary pattern, and predisposes carriers to a lifelong elevated low-density lipoprotein cholesterol (LDL-C) level. The prevalence of heterozygous FH has been estimated to be around one in 250 people [1], but it varies by age and geographic location. The prevalence of homozygous FH was around one in 200,000 to 300,000, and results in more severe and fatal atherosclerotic cardiovascular disease (ASCVD) if untreated [2].

In previous studies, FH was diagnosed clinically or genetically. The commonly used clinical criteria for FH are the Dutch Lipid Clinic Network (DLCN) algorithm, Simon Broome Register Group (SBRG) criteria, and Make Early Diagnosis to Prevent Early Death (MEDPED) criteria [3]. Presence of xanthoma or coronary heart disease (CHD), family history of CHD, and higher LDL-C level (exceeding 155-189 mg/dl) were the most common factors. Because only severe phenotypes were included in the clinical criteria, diagnosis of less severe FH cases might be missed. Low-density lipoprotein receptor (LDLR), apolipoprotein B (APOB), and proprotein convertase subtilisin/kexin 9 (PCSK9) are the three most common genes related to FH [4]. LDLR is the key mutant protein in both Western and Eastern countries, accounting for 80-85% of cases [4–6]. It has more than 3000 variants in the ClinVar database at present [6, 7]. There is still a small portion of patients who belong to the other genotypes, such as LDLRAP1 [4].

Accumulated LDL-C particles could trigger a sequence of oxidative processes, leading to atherosclerosis and arterial stenosis [6]. LDL-C is reported to confer both causal and accumulative risks for ASCVD throughout life [8]. By disturbing cholesterol metabolism over a person’s lifespan, FH increases the risk of premature ASCVD by at least 13- to 22-fold if left untreated [6]. The risk was still increased at least 3 times even with the same LDL-C level [9]. Previous lipid-lowering therapy studies showed a consistent result, whereby decreased LDL-C level lowered the risk of both ischemic stroke and major cardiovascular events [10]. However, whether FH increases the risk of stroke remains controversial. The Copenhagen study demonstrated for every 1 mmol/L increase of LDL-C level, the risk ratio of genetic cause was 1.45 for myocardial infarction, and 1.11 for ischemic stroke in FH cases [11]. One recent meta-analysis article stated genetically confirmed heterozygous FH did not seem to increase ischemic stroke risk, but peripheral arterial disease did [12]. In these two studies, only small subsets of FH mutations were considered, leading to selection bias. Therefore, we could not arbitrarily conclude a relationship between FH and stroke risk exists.

An Australian study showed the prevalence of clinically diagnosed FH was elevated to 19.4% in cardiovascular disease and to 11.5% in cerebrovascular disease, according to DLCN clinical criteria [13]. These values were much higher compared with the value of 0.4% in the general population. This suggests familial hypercholesterolemia might play an important role in coronary heart disease and in stroke. A Japanese study also revealed that FH frequency increased at least 5-fold in patients with acute coronary diseases compared with the general population by clinical diagnosis [14]. However, to the best of our knowledge, data on the prevalence of FH based on genetic evidence in patients with ischemic stroke is still lacking at present.

High LDL-C level was confirmed to be related to higher risk of cerebrovascular and cardiovascular diseases. However, a relationship between FH genes and the risk of ischemic stroke has yet to be established. Furthermore, most affected patients showed negative results for FH. We speculate that the prevalence of FH is higher in an ischemic stroke population based on pathological theory. Moreover, we attempted to explore the relationships between the subtypes of ischemic stroke and FH genes in our hospital cohort.

## RESULTS

### Description of the study groups

In total, we recruited 121 ischemic stroke patients, and divided them into two groups according to their LDL-C levels collected just after the index stroke event. There were 58 cases in the high-LDL group (LDL-C ≧ 130 mg/dl), and 63 cases in the low-LDL group (LDL-C < 130 mg/dl).

Based on LDL-C values clinically, high-LDL stroke patients were older, had a lower prevalence of previous CVA/CAD history, and the first stroke age was higher than in the low-LDL cases (Table 1). The two groups had similar percentages of hypertension, Peripheral Arterial Occlusive Disease (PAOD), Diabetes mellitus (DM), and smoking habit, as well as similar body mass index (BMI) levels, HbA1C values, and stroke severity (TOAST classification). Among the 58 cases in the high-LDL group, 6 carried pathogenic variants of FH which were all missense. Among the 63 cases in the low-LDL group, two variants of LDLR (rs777188764 and rs750474121) were identified in three patients as the “likely pathogenic variants”. Both of them were only discussed in a few case reports, with different results. Therefore, these two variants were defined as “potentially pathogenic” based on the ClinVar database. The demographic characteristics of these three patients are described in the Supplementary Table 1. Therefore, the prevalence of heterozygous FH was 10.3% (6/58) in ischemic stroke patients with a clinically higher LDL-C level, and was 5.0% (6/121) in all of our ischemic stroke patients.

**Table 1:** Characteristics of ischemic stroke patients with and without clinical hypercholesterolemia.

	**LDL<130 (n=63)**		**LDL≥130 (n=58)**		***P* value**
Gender					0.078
Female	15	(23.8%)	23	(39.7%)	
Male	48	(76.2%)	35	(60.3%)	
Age	55.0	(50.0-60.0)	62.5	(53.8-70.0)	<0.001**
LDL-C level	96.0	(81.0-115.0)	146.0	(138.3-164.3)	<0.001**
Body weight	70.1	(62.5-80.7)	66.9	(59.6-74.8)	0.161
BMI	25.6	(23.2-29.3)	25.1	(23.2-28.2)	0.750
TOAST classification:					
Large artery atherosclerosis	15	(23.8%)	12	(20.7%)	0.847
Small vessel occlusion	16	(25.4%)	20	(34.5%)	0.372
Cardiogenic	12	(19.0%)	8	(13.8%)	0.594
Unknown	10	(15.9%)	14	(24.1%)	0.362
TIA	10	(15.9%)	4	(6.9%)	0.209
Statin use (before stroke)	18	(28.6%)	9	(15.5%)	0.132
PAOD	4	(7.1%)	9	(18.0%)	0.160
Hypertension	39	(61.9%)	39	(67.2%)	0.673
DM	27	(42.9%)	21	(36.2%)	0.575
Fibrinogen (mg/dL)	311.7	(272.4-377.3)	300.4	(258.3-340.4)	0.235
D-dimer (mg/L FEU)	0.3	(0.2-0.4)	0.4	(0.2-0.7)	0.120
HbA1C	6.0	(5.7-7.4)	6.1	(5.8-6.9)	0.891
Intimal thickness (the thickest side, mm)	0.8	(0.6-0.9)	0.8	(0.7-0.9)	0.109
NIHSS (admission)	3	(1-6)	3	(1-4)	0.667
NIHSS (discharge)	1	(0-4)	1	(0-3)	0.912
mRS (admission)	2	(1-4)	2	(1-3)	0.171
mRS (discharge)	1	(1-3)	1	(1-2)	0.256
Hospitalization (days)	8.0	(5.8-11.0)	7.0	(5.0-9.0)	0.323
Smoking	24	(38.1%)	10	(21.7%)	0.107
Previous CVA or CAD history	23	(36.5%)	9	(15.5%)	0.016*
First stroke age	55.0	(49.0-60.0)	62.5	(53.0-69.8)	<0.001**

Chi-square test or †Mann-Whitney U test, Median (IQR). *p<0.05, **p<0.01.

BMI, body mass index; TIA, transient ischemic attack; CAD, coronary artery disease; PAOD, Peripheral Arterial Occlusive Disease; DM, Diabetes mellitus; HbA1C, glycated hemoglobin or Glycohemoglobin; IMT, intima-media thickness; NIHSS, NIH Stroke Scale; mRS, modified Rankin scale; CVA, cerebrovascular accident.

### Pathological variants of FH

**Figure 1:**
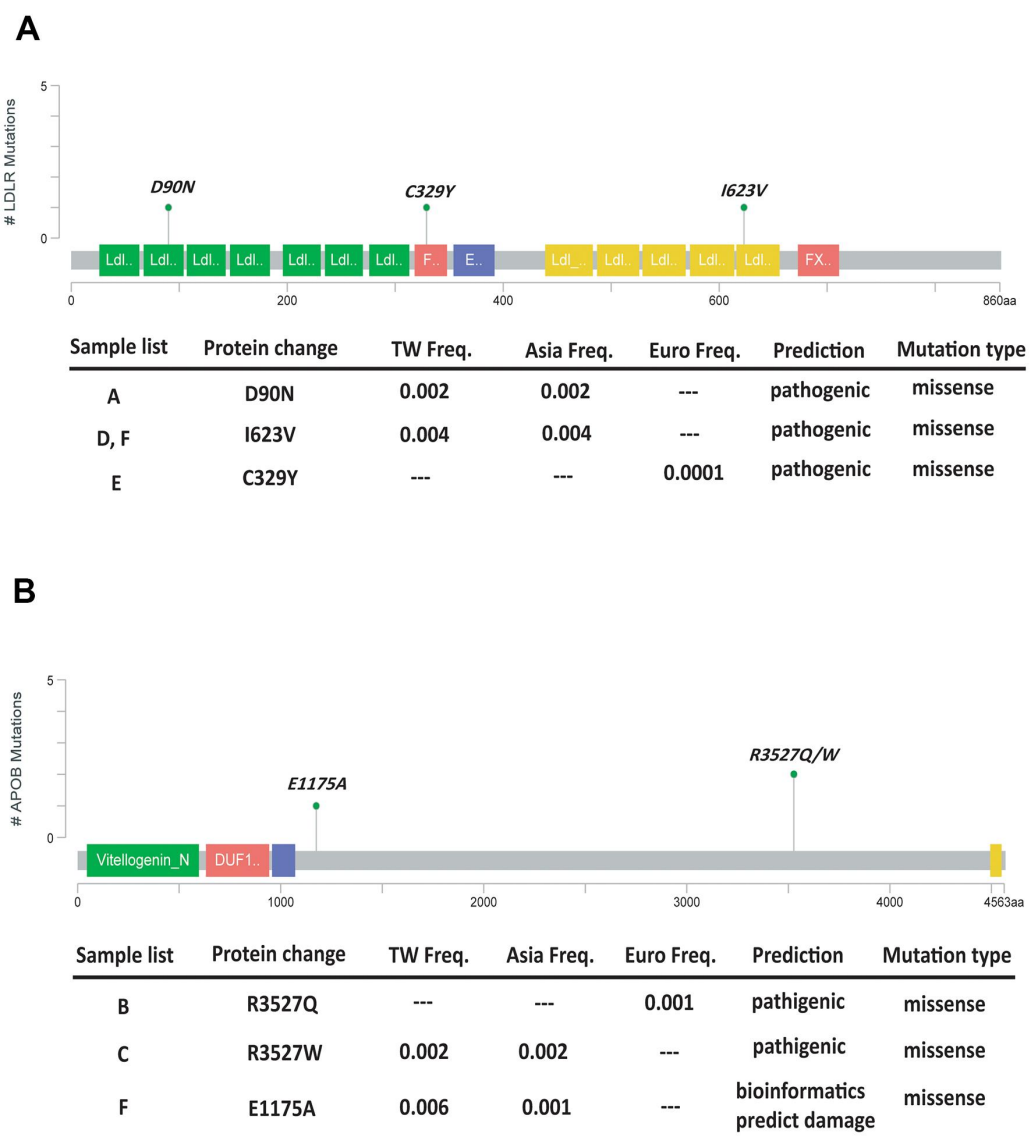
Lollipop plot to highlight our mutation patterns of *LDLR* (**A**) and *APOB* (**B**).

There were six pathogenic variants identified in 6 stroke patients with higher LDL-C level (Supplementary Table 2), and all resulted in a missense mutation. The located functional domains were depicted in Figure 1. Three of them were LDLR variants: c.268G>A, c.1867A>G, and c.986G>A. The other three were the APOB variants: c.10580G>A, c.10579C>T, and c.3524A>C. Only one variant, c.1867A>G, was relatively frequent in the database of Taiwan’s biobank, with a minor allele frequency of around 0.3%. The other variants were relatively rare in Taiwan’s population, with a minor allele frequency equal to or less than 0.1%. No pathogenic variants of *PCSK9* were identified in any subject. Among six patients, one carried two pathogenic variants located on *APOB* and *LDLR* genes. There were two cases carrying the same mutation of *LDLR*.

### Hypercholesterolemia with and without FH pathogenic variants

We compared the genetically diagnosed familial hypercholesterolemia cases with those having only clinical hypercholesterolemia without pathogenic variants. Stroke patients carrying the pathogenic variants demonstrated higher percentages of large artery atherosclerosis stroke subtype and transient ischemic attack (TIA), statin use before the index stroke, and previous history of cardiovascular or cerebrovascular diseases, as well as increased carotid intima-media thickness (IMT) (Table 2).

**Table 2:** Characteristics of ischemic stroke patients with clinically diagnosed hypercholesterolemia, with and without genetically verified familial hypercholesterolemia (FH).

	**Without FH (n=52)**		**With FH (n=6)**		***P* value**
Gender					0.072
Female	23	(44.2%)	0	(0.0%)	
Male	29	(55.8%)	6	(100%)	
Age	63.0	(55.0-69.0)	57.5	(48.8-74)	0.498
LDL level (mg/dL)	146.0	(136.0-159.5)	161.0	(150.5-179.0)	0.107
Body weight (kg)	66.7	(59.0-74.5)	73.5	(63.8-88.8)	0.198
BMI	25.1	(23.0-27.8)	25.6	(23.2-31.3)	0.537
TOAST classification:					
Large artery atherosclerosis	8	(15.4%)	4	(66.7%)	0.014*
Small vessel occlusion	20	(38.5%)	0	(0.0%)	0.084
Cardiogenic	8	(15.4%)	0	(0.0%)	0.581
Unknown	14	(26.9%)	0	(0.0%)	0.319
TIA	2	(3.8%)	2	(33.3%)	0.049*
Statin use (before stroke)	6	(11.5%)	3	(50.0%)	0.042*
PAOD	7	(15.2%)	2	(50.0%)	0.144
Hypertension	34	(65.4%)	5	(83.3%)	0.653
DM	18	(34.6%)	3	(50.0%)	0.657
Fibrinogen (mg/dL)	300.1	(258.2-332.6)	329.5	(264.4-452.5)	0.298
D-dimer (mg/L FEU)	0.4	(0.2-0.6)	0.3	(0.2-2.9)	0.948
HbA1C	6.2	(5.8-6.9)	5.9	(5.4-7.9)	0.466
Intimal thickness (the thickest side, mm)	0.8	(0.7-0.9)	0.9	(0.9-1.2)	0.024*
First event age (CVA or CAD)	63.0	(53.5-68.5)	56.5	(43.5-70.5)	0.399
Smoking	8	(19.5%)	2	(40.0%)	0.295
Previous CVA or CAD history	6	(11.5%)	3	(50.0%)	0.042*
First stroke age	63.0	(136.8-68.5)	57.5	(48.8-73.3)	0.548

Chi-square test or Fisher's exact test. †Mann-Whitney U test, Median (IQR). *p<0.05, **p<0.01.

### Double heterozygote

The characteristics of FH subjects are presented individually (Table 3). A wide-spectrum distribution of LDL-C level and onset age of cerebrovascular disease was found. All patients had increased carotid IMT, exceeding the generally accepted value of 0.8 mm [15].

**Table 3:** Characteristics and genetic information of individual FH cases.

**Patient number**	**Gender**	**Age of index stroke**	**BMI**	**Age of first stroke/TIA**	**Age of first CAD**	**Gene**	**Position**	**LDL at the index stroke (mg/dl)**	**statin use before index stroke**	**TOAST** **classification**	**IMT (mm)**	**mRS (initial→ 1 year)**
A	male	39	27.0	39	NA	LDLR	chr19:11213417	176	No	1	0.86	4→1
B	male	52	30.9	52	NA	APOB	chr2:21229160	154	No	1	0.99	4→0
C	male	61	24.2	61	NA	APOB	chr2:21229161	166	No	TIA	0.87	0→0
D	male	73	22.6	72	75	LDLR	chr19:11230789	140	Atorvastatin (20mg)	1	1.15	3→2
E	male	54	32.8	54	45	LDLR	chr19:11221373	156	Atorvastatin (10mg)	TIA	0.86	0→0
F	male	77	23.4	77	70	APOB	chr2:21238117	188	Atorvastatin (10mg)	1	1.15	5→5
						LDLR	chr19:11230789					

FH, familial hypercholesterolemia; BMI, body mass index; TIA, transient ischemic attack; CAD, coronary artery disease; IMT, intima-media thickness; mRS, modified Rankin scale; NA, not available (no event); chr, chromosome.

The only patient with double heterozygote of pathogenic *LDLR/APOB* variants (case F) showed more severe disease course and thickest IMT. He had a myocardial infarction at 70 years old, and recurrent stroke events at 77 years old. Compared with the other cases carrying pathogenic variants, he had the worst clinical status. He was diagnosed with vascular dementia before the age of 70, and his general health condition was considered to be approaching bedridden status at around the same time.

## DISCUSSION

This is the first study to explore the prevalence of familial hypercholesterolemia in Han-Chinese ischemic stroke and TIA patients using the NGS technique. We identified the prevalence of genetically diagnosed FH, and evaluated the clinical characteristics in clinically and genetically hypercholesterolemic stroke patients in comparison with other ischemic stroke patients.

### Prevalence of FH in ischemic stroke

In this study, we found the prevalence of heterozygous FH was at least 5.0% in all ischemic stroke and TIA patients. The prevalence further increased to 10.3% in those with an LDL-C level of 130 mg/dl and higher on the scene when stroke occurred. This result was more than 10 times higher than that in the general population according to data reported in previous epidemiological studies, from 0.3% based on the DLCN criteria to 0.46% with genetic confirmation [16]. In conclusion, the higher FH prevalence in stroke patients suggests that FH might be an important risk factor for ischemic stroke.

The diagnostic methods for FH vary in previous studies. Most used clinical criteria, and some used genetic confirmation. Furthermore, the methods of genetic tests also differed. Some used SNP array [11], in which only the genetic hotspots were identified. Others sequenced the specific genes [17], which might be more precise for identifying each variant. In our study, we used the NGS-based method to sequence the three target genes. Therefore, we could more accurately define the cases carrying FH genes, even in patients whose LDL-C level had been lowered by medication. The resultant hypercholesterolemia would not be masked by other causes, such as age and lifestyle. Therefore, we were able to more precisely determine the clinical characteristics that only arose as a result of genetic factors.

From a clinical perspective, an Austrian study showed the overall prevalence of potential FH (including probable/definite and possible FH) was 11.5% in stroke and TIA populations according to DLCN criteria [18], and increased to 19.4% in coronary heart disease [13]. With respect to genetics, the only study to be conducted on an ethnic Chinese population included a premature ASCVD group, which showed that the prevalence of genetically diagnosed FH was 4.4% (10 in 225 patients) [19]. The prevalence of genetically verified FH was lower than that of clinically diagnosed FH. This suggests other etiologies also result in hypercholesterolemia besides genes. However, subjects with genetic hypercholesterolemia have a relatively higher atherosclerotic risk than cases without [9], which was also reflected in the higher IMT values in our study.

To our knowledge, there are no data on the prevalence of genetically confirmed FH in ischemic stroke patients at present. Our result revealed there was one FH carrier for every 20 ischemic stroke cases, which was similar to the result for ASCVD in a study conducted in China [19]. The FH prevalence was even higher, that one carrier was identified for every 10 stroke cases with hypercholesterolemia. To date, although most recent studies focused on the effect of FH on cardiovascular events, our study demonstrated FH still confers a risk of ischemic stroke, a phenomenon that has been ignored. Therefore, due to the apparent high prevalence of FH, screening for FH to provide early identification and ischemic stroke prevention is warranted.

### FH and large artery atherosclerosis

Our result showed large vessel atherosclerosis stroke was the major stroke subtype in FH, compared with other hypercholesterolemia cases without FH carriers. Our data suggests that large vessel atherosclerosis is the most important subtype in FH-related ischemic stroke.

In contrast two other large studies conducted recently, the Norwegian register study [20] and the Copenhagen general population study [11], showed a negative result, indicating that FH did not have a causal relationship with any type of ischemic stroke. Another meta-analysis found the overall effect of heterozygous FH increased the risk of all kinds of ischemic stroke (p = 0.003). However, when the effect of genetically confirmed FH alone was analyzed, there was no significant association (p= 0.47) [12]. This might be because ischemic stroke was considered as one part, rather than as one of the subtypes, as in the present study.

Furthermore, another meta-analysis showed each mmol/L (39 mg/dL) decrease of LDL-C level lowered the relative risk of stroke by 21.1%. Intensive LDL-lowering therapy reduced fetal and non-fetal stroke events by 13% [10]. This suggests LDL still seems to be an important culprit in ischemic stroke. From the perspective of LDL-C pathophysiology, oxidized LDL-C triggers an inflammation cascade on the vessel wall, which facilitates plaque formation and atherosclerosis, leading to arterial stenosis [8]. The key process is atherosclerosis. Ischemic stroke comprises 5 subtypes according to TOAST classification [21], including large-artery atherosclerosis (LAA), cardioembolic, small vessel disease (SVD), undetermined, and others. Each subtype has a different etiology and pathogenesis, requiring different kinds of treatment [22]. Among the five subtypes of ischemic stroke, only the LAA subtype is mostly associated with the atherosclerotic process. LAA accounts for 25-60% of ischemic stroke [22, 23]. The other four etiologies are less strongly associated with LDL-related pathophysiology. When all stroke subtypes were combined in the analysis, the effect of LDL-related atherosclerosis was diluted.

In contrast, atherosclerosis is the main pathophysiological cause of coronary heart disease [24]. Therefore, both the Norwegian and Copenhagen studies found that an elevated risk of ischemic stroke was only found when FH cases had prior coronary heart disease. This result indirectly reflects the fact that our FH stroke patients had a higher percentage of previous stroke and ischemic heart disease events. Therefore, the generalized atherosclerosis-related risks would be increased in FH carriers. Consequently, it should be expected that FH carriers would not necessarily have an increased prevalence of all types of ischemic stroke. This supports our finding that aside from TIA, the most frequent ischemic stroke subtype in FH carriers was LAA.

### Carotid intima-media thickness (IMT) in FH

We found each FH carrier had an increased carotid IMT according to their age reference in our study. IMT is considered to be a marker of subclinical atherosclerosis, and is associated with common risk factors of stroke [25]. Increased IMT was reported to be related to elevated risk of all stroke types [26], and risk of the LAA type was relatively higher than that of the SVD subtype [25]. Increased IMT was reported in FH children as early as 8 years of age compared with their unaffected siblings [27]. In addition, calcium scores of coronary arteries measured by computed tomography was reported to be related to the patients’ IMT values. Rafal et al. also found the proximal carotid IMT values were better correlated with FH-related hypercholesteremia than with other etiologies [28]. Our study demonstrated a similar result: IMT was significantly increased in FH cases compared to non-FH cases under similar hyperlipidemic status. Therefore, we speculate that the application of carotid sonography to monitor IMT values may be useful for FH screening in clinical practice, even though it is not a clinically diagnostic criterion for FH.

### Double heterozygotes FH

Double heterozygotes for two different FH pathogenic genes had been reported [29], but the phenotypes and their severity have not been well described. In another case report, a subject had the phenotype severity of the double heterozygote ranking between homozygote and simple heterozygote [30]. In our study, the most severe FH case had the most elevated IMT, highest LDL level even under statin use, and highest mRS score compared with the other heterozygous FH cases. He had double heterozygote FH, carrying the *LDLR/APOB* pathogenic variants. We speculate that the number of pathogenic variants have a cumulative effect on their phenotype severity, leading to more morbidity in later life. Therefore, earlier and more aggressive intervention in double heterozygote FH is recommended. Moreover, further larger studies to identify the phenotypic spectrum of heterozygotes of FH are needed.

### Limitations

There were still some limitations in this study. First, the sample size was limited. Only 121 stroke patients were enrolled, and our FH stroke patients were all male. Second, we only sequenced the three most common genes related to FH in this study. Based on data presented in a previous report, around 5% of FH cases would not have been detected by our genetic panel [4, 31]. Third, we did not explore cognitive outcome after ischemic stroke in this study. In addition to LDL-C, ischemia itself might also trigger serial inflammatory processes in the brain [32]. Further cognitive evaluation in FH patients should also be considered in future research.

## CONCLUSIONS

The prevalence of heterozygous FH in Han-Chinese stroke patients was 5.0%, and was as high as 10.3% in those with the LDL-C of 130 mg/dl and higher. Stroke patients carrying FH pathogenic variants seemed to have a higher risk for large vessel stroke and TIA, which could be attributable to atherosclerotic pathogenesis. Moreover, the IMT exam could be a valuable tool to screen for FH in stroke patients with a high LDL-C level in order to identify at-risk individuals and apply early intervention.

## MATERIALS AND METHODS

### Participants

We enrolled ischemic stroke patients prospectively from the neurological ward or outpatient clinics of Taichung Veterans General Hospital from December 2018 to November 2019. Patients’ clinical characteristics were identified to classify the stroke subtype based on TOAST criteria. The LDL-C level was checked for this index stroke and the stroke severity using scores of the National Institute of Health Stroke Scale (NIHSS) and the Modified Rankin Scale, MRS (MRS). We excluded patients who refused to provide informed consent. Moreover, the clinical characteristics of the patients were recorded, including metabolic information and LDL-C levels. The carotid intima-media thickness (IMT) values were recorded from the thickest side.

First, we divided the patients into two groups according to an LDL-C level, which the threshold value was set at 130 mg/dl. Then, the genetic study was performed in both groups to identify the FH cases genetically.

This study was approved by the Ethics Committees of Taichung Veterans General Hospital (SE17355B).

### Genetic analysis

Genomic DNA was extracted using the QIAamp DNA Blood Mini Kit (QIAGEN, Germany) according to the manufacturer’s instructions. Next generation sequencing (NGS) was used to sequence the three target genes: *LDLR, APOB*, and *PCSK9*. The probes/primers specific for these genes were designed. Then, we used polymerase chain reaction (PCR) to amplify the candidate DNA fragments by QIAseq Targeted DNA Panels (QIAGEN) and sequenced them using the Illumina system. The overall analysis pipeline for somatic mutations is depicted in the Supplementary Figure 1. The Fastq files were uploaded to QIAGEN CLC Genomics Workbench (QIAGEN, Hilden, Germany) for read trimming, read alignment, and variant calling. The output VCF files were then uploaded to Base Space Variant Interpreter (Illumina) for annotations. Variants with a minimum coverage of 10 reads and Taiwan Biobank (TWB) allele frequency < 0.01 were kept.

The pathogenic or potentially pathogenic variants were determined by NCBI-ClinVar database (https://www.ncbi.nlm.nih.gov/pmc/). If the mutation was not found in these databases, the Scale-Invariant Feature Transform (SIFT) and Polymorphism Phenotyping v2 (Polyphen-2) (Harvard, Boston, MA, USA) were used to analyze conservation of the amino acid caused by the mutation; if the mutation was highly conserved among different species (SIFT score < 0.05 or Polyphen-2 score > 0.15), it was predicted to be a damaged mutation.

### Statistical analyses

SPSS version 20.0 (IBM, Armonk, NY, USA) was used for all statistical analyses. The Kolmogorov-Smirnov test was used to confirm that the characteristics of these groups did not present a normal distribution. A *p* value of less than 0.05 was defined as statistically significant.

## Supplementary Material

Supplementary Figure 1

Supplementary Tables
