# Supplementary material for: Characterization of familial hypercholesterolemia in Taiwanese ischemic stroke patients
Source: Aging (Albany NY). 2021 Jul 27;13(15):19339–51. doi: 10.18632/aging.203320 (PMC8386562; doi:10.18632/aging.203320)
Supplement: Supplementary Figure 1 [file aging-13-203320-s001.pdf]

SUPPLEMENTARY FIGURE

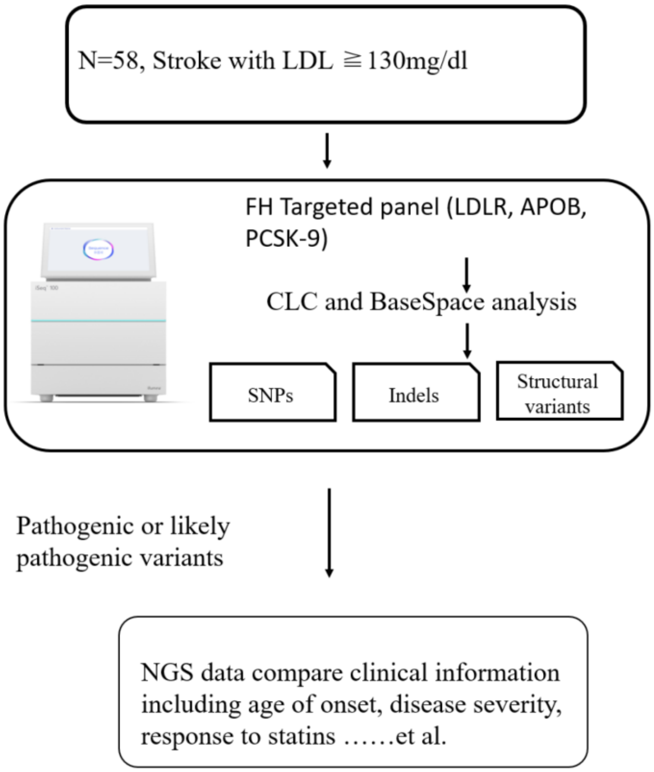

Supplementary Figure 1. Flow chart of next-generation sequencing procedure for three familial hypercholesterolemia genes (LDLR, APOB, PCSK-9).
