# Supplementary material for: Characterization of familial hypercholesterolemia in Taiwanese ischemic stroke patients
Source: Aging (Albany NY). 2021 Jul 27;13(15):19339–51. doi: 10.18632/aging.203320 (PMC8386562; doi:10.18632/aging.203320)
Supplement: Supplementary Tables [file aging-13-203320-s002.pdf]

SUPPLEMENTARY TABLES

Supplementary Table 1. The detailed characteristics of the cases with likely pathogenic variants of FH, who were in the low-LDL group.

Pathological variant carrier	G	H	I
Gender	M	M	M
BMI	19	21.4	24.7
Age of first stroke/TIA	56	51	60
Age of first CAD	56	51	60
Age of this index stroke	56	55	60
Gene	LDLR	LDLR	LDLR
Position	chr19:11227550	chr19:11227550	chr19:11213441
rs number	rs777188764	rs777188764	rs750474121
Mutation type	missense	missense	missense
LDL level at the index stroke	103	45	122
Statin use before index stroke	No	Yes	No
TOAST classification	cardiogenic	cardiogenic	cardiogenic
IMT (mm)	0.52	0.82	0.75
IMT limits based on age	0.72	0.8	0.8
NIHSS (admission→ discharge)	6→0	10→10	7→6
mRS (initial→ 1 year)	2→1	4→3	4→4
DM	0	1	0
Hypertension	0	1	0
Tendon xanthomas	0	0	0
Eyelid xanthelasmas	0	0	0
Vascular dementia	No	No	No

BMI, body mass index; TIA, transient ischemic attack; CAD, coronary artery disease; IMT, intima-media thickness; NIHSS, NIH Stroke Scale; mRS, modified Rankin scale.

Supplementary Table 2. The detailed characteristics of the FH carriers, who were all in the high-LDL group.

Pathological variant carrier	A	B	C	D	E	F
**Gender**	M	M	M	M	M	M
**BMI**	27	30.9	24.2	22.6	32.8	23.4
**Age of first stroke/TIA**	39	52	61	72	54	77
**Age of first CAD**	not occur	not occur	not occur	75	45	70
**Age of this index stroke**	39	52	61	73	54	77
**Gene**	LDLR	APOB	APOB	LDLR	LDLR	APOB
**Position**	chr19:11213417	chr2:21229160	chr2:21229161	chr19:11230789	chr19:11221373	chr2:21238117, chr19:11230789
**rs number**	rs749038326	rs5742904	rs144467873	rs555292896	rs761954844	rs1307980669, rs555292896
**Mutation type**	missense	missense	missense	missense	missense	missense
**LDL level at the index stroke**	176	154	166	140	156	188
**Statin use before index stroke**	No	No	No	Atorvastatin(20mg)	Atorvastatin (20mg)	Atorvastatin (10mg)
**TOAST classification**	large artery atherosclerosis	large artery atherosclerosis	TIA	large artery atherosclerosis	TIA	large artery atherosclerosis
**IMT (mm)**	0.86	0.99	0.87	1.15	0.86	1.15
**IMT limits based on age**	0.72	0.8	0.8	0.8	0.8	0.8
**NIHSS (admission → discharge)**	6→4	5→3	1→0	2→1	0→0	6→5
**mRS (initial→ 1 year)**	4→1	4→0	0→0	3→2	0→0	5→5
**DM**	0	1	0	1	0	1
**Hypertension**	1	1	1	0	1	1
**Tendon xanthomas**	0	0	0	0	0	0
**Eyelid xanthelasmas**	0	0	0	0	0	0
**Vascular dementia**	No	No	No	No	No	Yes

BMI, body mass index; TIA, transient ischemic attack; CAD, coronary artery disease; IMT, intima-media thickness; NIHSS, NIH Stroke Scale; mRS, modified Rankin scale.
